# Phase III randomized trial of preoperative concurrent chemoradiotherapy versus preoperative radiotherapy for patients with locally advanced head and neck squamous cell carcinoma

**DOI:** 10.18632/oncotarget.15107

**Published:** 2017-02-05

**Authors:** Junlin Yi, Xiaodong Huang, Zhengang Xu, Shaoyan Liu, Xiaolei Wang, Xiaohui He, Dehong Luo, Jingwei Luo, Jianping Xiao, Shiping Zhang, Kai Wang, Yuan Qu, Yuan Tang, Weixin Liu, Guozhen Xu, Li Gao, Dian Wang

**Affiliations:** ^1^ Department of Radiation Oncology, National Cancer Center/Cancer Hospital, Chinese Academy of Medical Sciences and Peking Union Medical College, Beijing, P. R. China; ^2^ Department of Surgery, National Cancer Center/Cancer Hospital, Chinese Academy of Medical Sciences and Peking Union Medical College, Beijing, P. R. China; ^3^ Department of Medical Oncology, National Cancer Center/Cancer Hospital, Chinese Academy of Medical Sciences and Peking Union Medical College, Beijing, P. R. China; ^4^ Department of Diagnostic Radiology, National Cancer Center/Cancer Hospital, Chinese Academy of Medical Sciences and Peking Union Medical College, Beijing, P. R. China; ^5^ Department of Radiation Oncology, Rush University Medical Center, Chicago, USA

**Keywords:** head and neck squamous cell carcinoma, preoperative radiotherapy, concurrent chemoradiotherapy, multimodality treatment, organ function preservation

## Abstract

**Purpose:**

To determine the role of preoperative concurrent chemoradiotherapy in the treatment of locally advanced head and neck squamous cell carcinoma (HNSCC).

**Methods:**

A total of 222 patients with stage III/IVA-B HNSCC were randomly assigned to receive preoperative concurrent chemoradiotherapy (Pre-S CRT, weekly cisplatin 30mg/m^2^) or preoperative radiotherapy alone (Pre-S RT). Survival analysis was estimated by the Kaplan-Meier method and compared by the log-rank test.

**Results:**

With a medial follow-up of 59 month, the 5-year overall survival (OS), progression-free survival (PFS), distant metastasis-free survival (DMFS) of Pre-S CRT *v* Pre-S RT group were 53.8% *v* 39.0% (hazard ratio [HR], 0.74, 95% CI, 0.50 to 1.10, *P* = 0.13), 53.2% *v* 38.7%, (HR, 0.69, 95% CI, 0.47 to 1.01, *P* =0.06), and 80.4% *v* 68.1% (HR, 0.53, 95% CI, 0.28 to 0.98, *P* = 0.04), respectively. In patients with larynx-hypopharynx primaries, the 5-year OS, PFS and DMFS of Pre-S CRT *v* Pre-S RT were 62.7% *v* 38.8% (HR, 0.59, 95% CI 0.35 to 1.02, *P* = 0.054), 63.1% *v* 39.9% (HR, 0.52; 95% CI 0.30 to 0.89, *P* = 0.03) and 86.2% v 63.3% (HR, 0.35, 95% CI 0.15 to 0.82, *P* = 0.01), respectively.

**Conclusion:**

The addition of weekly cisplatin concurrent to preoperative RT does not improve OS, but improve DMFS in locally advanced HNSCC. However, in a subset of patients with the larynx-hypopharynx primaries, preoperative chemoradiotherapy has significantly improved PFS and DMFS, and has also provided a borderline benefit in OS in comparison with preoperative radiotherapy alone.

## INTRODUCTION

Although preoperative radiotherapy *(RT)* was reportedly associated with more toxicity than postoperative RT by the results of RTOG multicenter phase 3 trial 73-03 [1–3], preoperative RT strategy has been successfully utilized with acceptable toxicity profile in our cancer center [4–10]. We have routinely prescribed preoperative RT (50 Gy in 25 fractions) to locally advanced HNSCC, followed by either resection of non-responding tumor (defined as less than 80% shrinking) or additional RT boost to other responding tumors. Benefits of this preoperative treatment strategy including organ-function preservation and avoiding surgical related morbidities such as poor quality of life outcomes (functional and cosmetic) for patients who had good response to preoperative radiotherapy and could cured by radical radiotherapy; and might facilitate resection for tumors that does not respond to preoperative RT and improved outcomes of these patients. Toxicities associated with preoperative radiotherapy were reported to be not *higher than* those associated with postoperative radiotherapy in our experience.

This phase 3 study was developed in early 2000 when results of several phase 2 studies suggested that preoperative concurrent chemoradiotherapy (CRT) resulted in excellent treatment outcome with pathological complete response rate of 35%-61% and 5-years overall survival rate of 70%-81.5% in patients with non-nasopharyngeal HNSCC [11–18]. A subsequent meta-analyses data showed that CRT can improve the overall survival with a 6.5% to 8% absolutely benefit at 5 years, compared with radiotherapy alone [19]. Accumulated data including results of several phase 3 trials and updated meta-analysis have indeed demonstrated survival benefits of definitive CRT over definitive RT alone [20, 21]. Given the established experience of preoperative radiotherapy of locally advanced non-nasopharynx HNSCC in our institution, we launched this phase 3 trial to investigate whether or not the preoperative concurrent CRT is superior to preoperative RT in the treatment of locally advanced HNSCC. For patients with significant response (80% or more shrinking of primary tumor) to initial 5-week RT or 5-week CRT, we chose to proceed with definitive RT *versus* CRT in this protocol.

## RESULTS

### Clinical features

From September 2002 to May 2012, a total of 240 patients were enrolled to this study, with 116 patients assigned to the Pre-S CRT group and 124 patients to the Pre-S RT group. In the Pre-S CRT group, 2 patients withdraw from information consent, 4 patients did not finish protocol treatment because of financial reason, 6 patients lost follow-up immediately after treatment. In the Pre-S RT group, 2 patients did not finish protocol treatment because of financial reason, 4 patients lost follow-up immediately after treatment. A total Of 222 patients were evaluable, 104 patients in the Pre-S CRT group and 118 patients in the Pre-S RT group. The clinical characteristics of these two groups were summarized in the Table 1.

**Table 1:** Patient characteristics in Pre-S CRT and Pre-S RT groups

Characteristics	Pre-S CRT (*n* = 104)		Pre-S RT (*n* = 118)		*P*
	*n*	%	*n*	%	
Gender Male Female	85 19	81.7 18.3	105 13	82.2 17.8	0.13
Medial age (years)		55	55		
Primary site Oral cavity oropharynx Hypopharynx/ Larynx	14 30 60	13.5 28.8 57.7	14 36 68	11.9 30.5 57.6	0.52
T stage T1 T2 T3 T4a T4b	6 18 22 51 7	5.8 17.3 21.2 49.0 6.7	6 25 43 36 8	5.1 21.2 36.4 30.5 6.8	0.05
N stage N0 N1 N2 N3	9 20 62 13	8.7 19.2 59.6 12.5	19 22 60 17	16.1 18.6 50.9 14.4	0.36
Clinical group III IVA IVB	14 70 20	13.5 67.3 19.2	21 73 24	17.8 61.9 20.3	0.62
Medial Chemotherapy cycle		5	none		
Radiotherapy technique 3DCRT IMRT	62 42	59.6 40.4	62 56	52.5 47.5	0.29

Pre-S RT: preoperative radiotherapy; Pre-S CRT: preoperative concurrent chemoradiotherapy;

3DCRT: three-dimensional conformal radiotherapy; IMRT: intensity-modulated radiotherapy.

### Response to preoperative treatment

In the Pre-S RT group, 70.3% (83/118) patients were responders while 29.7% (35/118) patients were non-responders. Similarly, in the Pre-CRT group, 64.4% (67/104) patients were responders while 35.6% (37/104) were non-responders (*P* = 0.35). For the non-responders who underwent resection of primary tumors, the pathological complete responses rates were 42.9% (15/35) in the Pre-S RT *versus* 27.0% (10/37) in the Pre-S CRT group (*P* = 0.16).

### Clinical outcomes

With a medial follow-up of 59 month in a range of 7 to 122 months, the 5-year estimated LRC, OS, progression free survival (PFS), and distant metastasis free survival (DMFS) for the entire group were 66.4%, 46.2%, 45.6% and 73.9%, respectively. The 5-year DMFS and PFS of the Pre-S CRT *v* Pre-S RT groups were 80.4% *v* 68.1% (HR, 0.53, 95% CI, 0.28 to 0.98, *P* = 0.04) and 53.2% *v* 38.7% (HR, 0.69, 95% CI, 0.47 to 1.01, *P* = 0.06), respectively. However, there was no difference in the 5-year OS and LRC rates between the Pre-S CRT and Pre-S RT, 53.8% *v* 39.0%, (HR, 0.74, 95% CI, 0.50 to 1.10, *P* = 0.13) for OS and 70.1% *v* 62.4% (HR, 0.83, 95% CI, 0.50 to 1.38, *P* = 0.47) for LRC, respectively (Figure 2).

**Figure 1:**
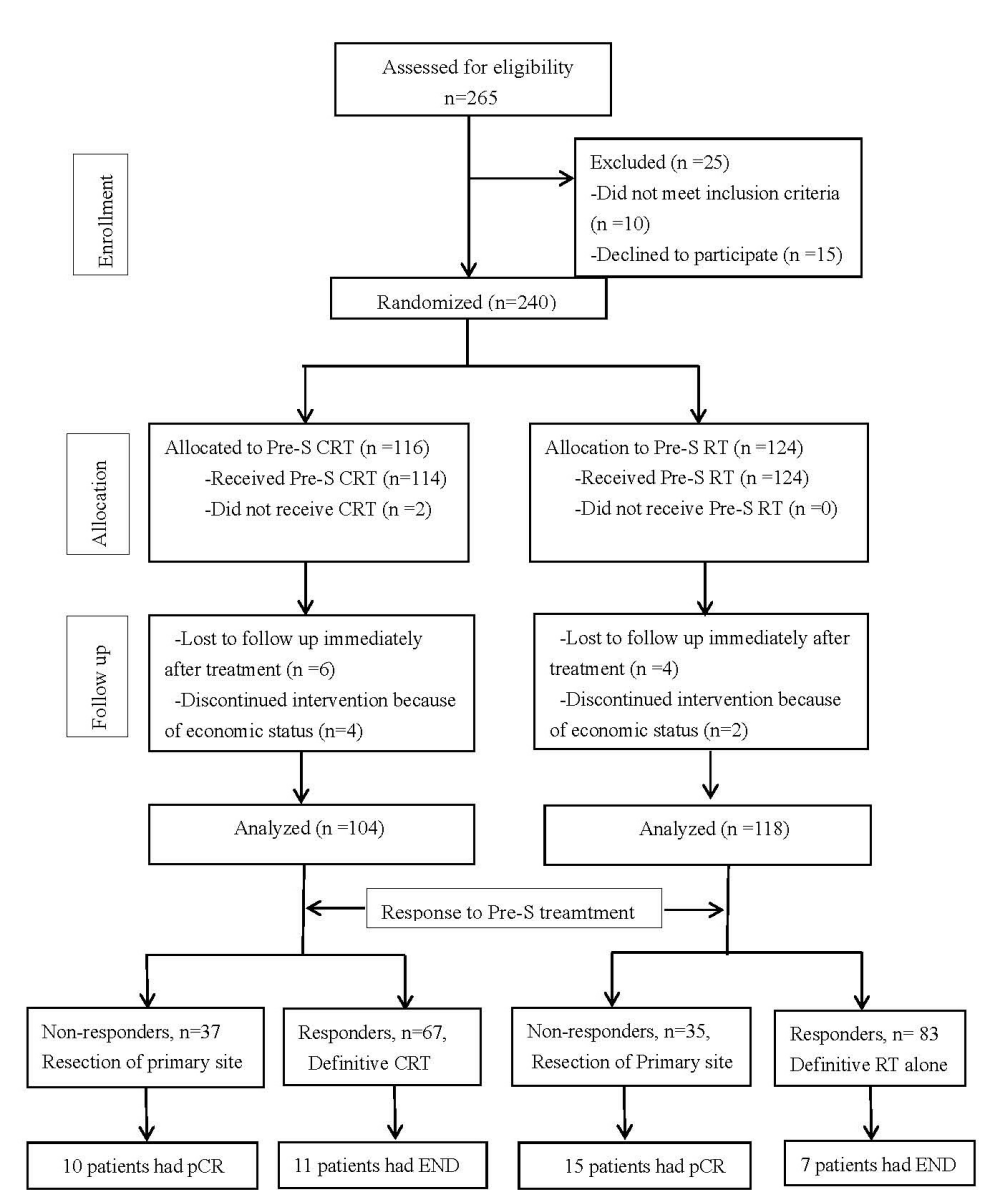
CONSORT diagram

**Figure 2:**
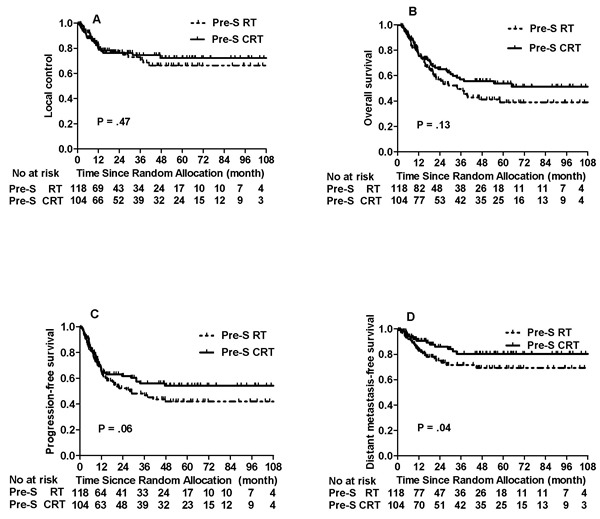
Comparison of the treatment outcomes between Pre-S RT and Pre-S CRT groups for whole cohort

### Patterns of failure

One hundred and eight patients developed disease recurrence including failures in primary sites, regional nodal and distant metastasis. Of them were 44 in the Pre-S CRT and 64 in the Pre-S RT group. Failures in primary site or regional neck or distant metastasis were not different between these two groups as shown in Table 2.

**Table 2:** Failure pattern in the Pre-S CRT and Pre-S RT groups

Failure pattern	Treatment				*P*
	Pre-SCRT (*n* = 104)		Pre-S RT (*n* = 118)		
	*n*	%	*n*	%	
Primary site	27	25.9	34	28.8	0.635
Regional lymph node	10	9.62	12	10.2	0.890
Distant	15	14.4	29	24.5	0.058

Pre-S RT: preoperative radiotherapy; Pre-S CRT: preoperative concurrent chemoradiotherapy.

### Treatment-related toxicities

The main hematology toxicities observed in this trial were leucopenia, anemia, and thrombocytopenia. There was a significant difference in grade 3+ hematology toxicities between Pre-S CRT and Pre-S RT group, 16.3% *v* 0.8% (*P* < 0.001). The main non-hematology toxicities were mucositis, pharygalgia, xerostomia, and skin reactions. There were no significant differences in rates of the above main non-hematology toxicities between the CRT and RT groups (Table 3). The most common surgery-related complications were pharyngeal fistula, wound infection and wound healing delay. There were no significant difference between Pre-S CRT and Pre-S RT group, with pharyngeal fistula incidence of 5 (5/37) and 4(4/35), wound healing delay and infection incidence of 2 (2/37) and 1(1/35)in Pre-S CRT and Pre-S RT group, repectively.

**Table 3:** Incidences of treatment-related toxicities in the Pre-S CRT and Pre-S RT groups

Acute Toxicities	Pre-S CRT (*n* = 104)				Pre-S RT (*n* = 118)				*P*
	Grade I/II		Grade III/IV		Grade I/II		Grade III/IV		
	*n*	%	*n*	%	*n*	%	*n*	%	
Anemia	46	44.2	1	0.96	19	16.1	0	0	<0.001
Leucopenia	74	71.2	11	10.6	40	33.9	0	0	<0.001
Thrombocytopenia	19	18.3	5	4.8	3	2.55	1	0.85	<0.001
Liver function	3	2.9	0	0	6	5.1	0	0	0.41
Renal function	5	4.81	1	0.96	2	1.7	0	0	0.23
Mucositis	74	71.2	29	27.9	99	83.9	25	21.2	0.37
Pharyngalgia	82	78.8	14	13.5	99	83.9	13	11.0	0.87
Xerostomia	103	99.0	1	0.96	102	86.5	0	0	0.88
Skin reaction	94	90.4	6	5.8	114	96.6	4	3.39	0.15

Pre-S RT: preoperative radiotherapy; Pre-S CRT: preoperative concurrent chemoradiotherapy.

### Subset analyses restricted to larynx-hypopharynx primaries

**Figure 3:**
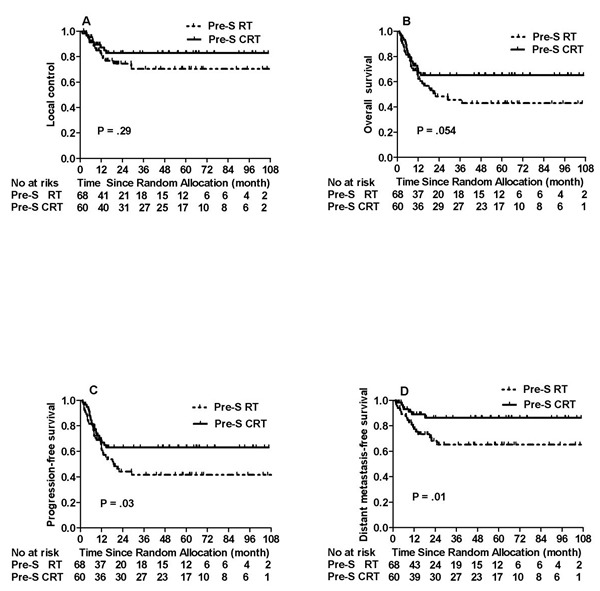
Comparison of the treatment outcomes between Pre-S RT and Pre-S CRT groups ristricted to primary larynx-hypopharynx carcinoma only

Of 128 patients with larynx-hypopharynx primaries enrolled to this study, 60 (57.6%) patients were in the Pre-S CRT while 68 (57.7%) patients were in the Pre-S RT group. The 5-year OS of Pre-S CRT *v* Pre-S RT group shows a trend toward a statistical difference, 62.7% *v* 38.8% (HR, 0.59, 95% CI, 0.35 to 1.02, *P* = 0.054). The 5-year PFS, DMFS and LRC between Pre S RT *v* Pre S CRT were 63.1% *v* 39.9% (HR, 0.52, 95% CI, 0.30 to 0.89, *P* = 0.03) for PFS, 86.2% *v* 63.3% (HR, 0.35, 95% CI, 0.15 to 0.82, *P* = 0.01) for DMFS and 81.4% *v* 69.3% (HR, 1.59, 95% CI, 0.71 to 3.54, *P* = 0.29) for LRC. The laryngectomy-free survival in the Pre-S CRT and the Pre-S RT group were 75.5% and 64.5%, (HR, 0.64, 95% CI, 0.30 to 1.36, *P* *=* 0.23) (Figure 3).

## DISCUSSION

To our best knowledge, this is the first phase 3 trial to compare preoperative chemoradiotherapay (CRT) and preoperative RT in the treatment of non-nasopharynx HNSCC. Results of this phase 3 trial show that the addition of weekly 30 mg/m^2^ cisplatin to preoperative radiotherapy did not enhance OS, but significantly improved 5-year DMFS, 80.4% *v* 68.1% (*P* *=* 0.04), and also borderline significance in PFS (53.2% *v* 38.7%, *P =* 0.06) when compared to preoperative radiotherapy alone. In a subset of the larynx-hypopharynx primaries, the addition of concurrent chemotherapy to preoperative radiotherapy have significantly improved the PFS, DMFS, and even the OS with borderline significance (*P* = 0.054).

We have not observed a statistical difference in 5-year OS between the Pre-S CRT and Pre-S RT (53.8% *v* 39.0%, *P* *=* 0.13) in the entire group of non-nasopharynx HNSCC, however, the OS rates of these two groups are not different from results of the previous phase 3 trials of comparing definitive CRT *versus* definitive RT alone in the North America and Europe. For example, Adelstein et al [14] reported 5-year OS of 50% for concurrent CRT group and 48% for RT alone group in patients with locally advanced HNSCC. Forastiere et al [22] reported the 5-year OS were 56% for RT alone group and 54% for concurrent CRT group (RTOG 91-11 trial). In addition, similar OS results were also reported in patients with locally advanced HNSCC treated with concurrent CRT *versus* induction chemotherapy followed by concurrent CRT. Lefebvre et al [23] reported no difference in 5-year OS between patients treated with induction chemotherapy followed by concurrent CRT with or without surgery *versus* patients treated with concurrent CRT with or without surgery in locally advanced larynx-hypopharynx carcinomas (48.5% *v* 51.9%, *P* = 0.446). Most recently, Lorch et al [24] reported significant improvement in 5-year OS in patients treated with intensive neoadjuvant induction chemotherapy consisting of docetaxel, cisplatin and 5-flurouracial (TPF) followed by concurrent CRT compared with patients treated with induction chemotherapy consisting of cisplatin and 5-flurouracial (PF) followed concurrent chemoradiotherapy (52% *v* 42%, *P* = 0.014). It is noted that 5-year OS (52%) in patients treated with induction TPF followed by concurrent chemoradiotherapy in that study is not different from OS (53.8%) observed in the Pre-S CRT group of our study and the OS results of other previous phase III trials using definitive concurrent CRT [14, 22].

Currently three cycles of high-dose cisplatin 100 mg/m^2^ every three weeks are commonly used in combination with definitive radiotherapy of 70 Gy in 35 daily fractions in the treatment of locally advanced HNSCC in many institutions of the North America. However, this high-dose cisplatin regimen concurrent with definitive RT is reportedly very toxic, only 31% patients were able to complete the planed full-dose cisplatin [25]. Severe toxicities (grade 3 or more) of this high-dose cisplatin regimen concurrent with definitive RT of HNSCC were much higher compared with RT alone. Results from definitive concurrent CRT arm of three RTOG HNSCC trials (RTOG 91-11, 97-03, and 99-14) showed that 43% patients had a severe late toxicity, and 10% patients died from treatment-related late toxicities. The factors associated with these severe late toxicities include old age, advanced T-stage, and larynx-hypopharynx primaries, neck dissection after definitive CRT [26]. Considering the poor tolerance of Chinese patients, increased percentage of patients with larynx-hypopharynx primaries, and common use of planned dissection for patients with N2/3, we chose this weekly 30mg/m^2^ cisplatin regimen concurrent with preoperative RT. Results of this study indeed show that this is well tolerated CRT regimen, most of toxicities were grade 1 and grade 2, with only 16.3% of grade 3+ hematology toxicities observed in Pre-S CRT group (Table 2). We did not observe difference in non-hematologic toxicities between these two groups. More recently, this weekly cisplatin regimen (30-40mg/m^2^) concurrent with RT was chosen to treat Asian patients with locally advanced HNSCC [27].

There are several possible reasons to explain why addition of weekly Cisplatin concurrent to preoperative RT failed to provide benefits in overall survival. One pitfall might be that a mixed population of patients with HNSCC including oral cavity, oropharynx and larynx-hypopharynx primaries was enrolled to this study. Tumor biology and aggressiveness of the above subsites might be different event though their squamous cell cancer histology is the same. Our subset analyses of patients with larynx-hypopharynx primaries indeed demonstrated that the addition of weekly cisplatin to preoperative RT has significantly improved PFS, DMFS, and also borderline OS (might be secondary to a limited sample size of larynx-hypopharynx primaries enrolled to this study). Another observation is that a high percentage (57.7%) of larynx-hypopharynx primaries was recruited to this study. This is significantly different from previous studies in North America and Europe where oropharynx primary was dominant. This might translate to a significant difference in tumor biology and treatment response to CRT or RT. For example, HPV is prevalent in oropharynx primary in North America and Europe and the HPV positive oropharynx cancer is known to be highly sensitive to CRT or RT. The HPV positive oropharynx cancer patients had much better overall survival compared with the HPV negative HNSCC [28]. Chemotherapy might not even be required for oropharynx cancer with favorable risk factors [29, 30].

For locally advanced larynx-hypopharynx primaries, larynx function preservation is another objective goal. Results of the subset analyses showed the laryngectomy-free survival in the Pre-S CRT is higher than that in the Pre-S RT group, 75.5% *v* 64.5%, but without statistical significance (*P* = 0.23). However, this result is not significantly different from literature reports, for example, Lefebvre et al [23 ]reported 3-year and 5-year estimates of retaining a functional larynx in patients treated in the induction-chemotherapy arm were 39.5% and 30.5%. Pointreau et al [31] reported a the 3-year actuarial larynx preservation rate was 70.3% with TPF vs 57.5% with PF chemotherapy (*P* = 0.03) in patients with resectable larynx- hypopharynx primaries.

In summary, the addition of weekly cisplatin concurrent to preoperative RT has not provided overall survival benefit in the treatment of locally advanced non-nasopharynx HNSCC (a mixed population of oral cavity, oropharynx, hypopharynx and larynx) in this study, however, results from the subset analysis of larynx-hypopharynx primaries dominant in this study (57.7%) suggest the addition of weekly cisplatin has provided significant benefits in progression-free survival, distant metastasis free survival, and borderline benefit in overall survival, along with acceptable toxicity profile, compared with preoperative RT alone.

## MATERIALS AND METHODS

### Eligibility criteria

Adult patients with resectable, histologically proven squamous cell carcinoma of oropharynx, hypopharynx, oral cavity and larynx, stage III to IVB according to the 2002 UICC/AJCC staging system, Karnofsky performance status score ≥70, adequate hematologic (leukocyte count>4,000/mm^3^ and platelet count >100,000/mm^3^), normal renal function (serum creatinine level>1.5 mg/dL), and normal hepatic function were eligible for this study. Exclusion criteria include a previous history of chemotherapy or radiotherapy, any other cancer diagnosis within 5 years and severe comorbidities that contraindicated the use of chemotherapy or radiotherapy.

All patients had history and physical examination including the dedicated head and neck examination, and endoscopy evaluation of upper aero-digest tract, CT and/or MRI of neck, chest X-ray, CT scan or ultrasonic of abdomen and pelvis before the registration.

### Random assignment and treatment

This is an open-labeled randomized phase 3 trial approved by our institution human study board. Each eligible patient first signed informed consent, were then registered and randomly assigned to receive either preoperative concurrent chemoradiotherapy (Pre-S CRT group), or preoperative radiotherapy (Pre-S RT group). One stratification factor was primary disease site (larynx-hypopharynx v. other). The detailed study design information was shown in Figure 1.

For patients assigned to the CRT group, cisplatin of 30 mg/m^2^ once per week was delivered at the first day of radiotherapy. The initial radiotherapy regimen in both arms was identical, preoperative dose of 50 Gy in 25 daily fractions prescribed to the primary tumor and nodal disease. The radiotherapy technique was either conventional radiotherapy or recently intensity-modulated radiotherapy (IMRT) using 6 MV photons.

### Assessment of tumor response and surgery decision making

The tumor response was assessed at the end of 5^th^ week (50 Gy) by CT and/or MRI and endoscopy examination. All cases were then discussed in our weekly multidisciplinary head and neck oncology board. Responders (≥80% reduction of primary lesion) received a boost to primary gross tumor volume up to a total dose of 70 Gy in 35 daily fractions in Pre-S RT group or in combination with concurrent weekly cisplatin (Pre-S CRT). A modified neck dissection was planned for all patients with N2/3 within 6-8 weeks after the completion of 70 Gy. Non-responders ( < 80% reduction of primary lesion) underwent resection of primary tumor and modified neck dissection in 4-6 weeks after the completion of preoperative 50 Gy in Pre S RT group or in combination with concurrent weekly cisplatin in Pre S CRT group.

### Follow-up and statistics analysis

The first follow-up visit was done at 1 month after the completion of protocol treatment, and then every 3 month for the first 2 years, every 6 month for the 3-5 years, and then once a year.

The primary endpoint of this study was overall survival (OS), defined as the time to death as a result of any cause. Assuming that the 5-year OS in the Pre-S RT group was 40% and 55% in the Pre-S CRT group, with a two-sided log-rank test at a level of significance of 0.05 and 80% power to detect the differences, with an estimated rate of early dropout or loss to follow-up of 20%, the target accrual sample size was 210 patients.

Secondary endpoints included locoregional control (LRC), progression-free survival, distant metastasis-free survival and laryngectomy-free survival for larynx- hypopharynx primary. All events were measured from the date of registration and the analyses were performed on an actual-to-treat basis to compare the treatment outcomes between these two groups.
